# Retraction Note: The anti-proliferative and anti-inflammatory response of COPD airway smooth muscle cells to hydrogen sulfide

**DOI:** 10.1186/s12931-021-01816-7

**Published:** 2021-08-25

**Authors:** Mark M. Perry, Bernadett Tildy, Alberto Papi, Paolo Casolari, Gaetano Caramori, Karen Limbert Rempel, Andrew J. Halayko, Ian Adcock, Kian Fan Chung

**Affiliations:** 1 School of Pharmacy & Biomedical Sciences, University of Portsmouth, St. Michael’s Building, White Swan Road, Portsmouth, PO1 2DT UK; 2 Airways Disease, National Heart and Lung Institute, Imperial College, London & Royal Brompton NIHR Biomedical Research Unit, London, SW3 6LY UK; 3 Sezione di Medicina Interna e Cardiorespiratoria, Centro Interdipartimentale per lo Studio delle Malattie Infiammatorie delle Vie Aeree e Patologie Fumo-Correlate (CEMICEF, formerly termed Centro di Ricerca su Asma e BPCO), Università di Ferrara, Ferrara, Italy; 4 Unità Operativa Complessa diPneumologia, Dipartimento di Scienze BiomedicheOdontoiatriche e delle Immagini Morfologiche e Funzionali (BIOMORF), Università degli Studi di Messina, Messina, Italy; 5 Departments of Internal Medicine & Physiology, Respiratory Hospital, Sherbrook Street, Winnipeg, MB R3A 1R9 Canada

## Retraction to: Respiratory Research (2018) 19:85 https://doi.org/10.1186/s12931-018-0788-x

The Editors-in-Chief have retracted this article. After publication concerns were raised about two of the figures, specifically:

- In Figure 3a, the MPST blot for non-smokers appears to be the same as the CBS blot for smokers.
- In Figure 5a, the beta-actin blot for smokers appears to be the same as the beta-actin blot for smokers in Figure 3c in a previous article [1].

An investigation by Imperial College into the integrity of these images was unable to reach a conclusion as it was established that the raw data and images from this study are not available for examination; it was therefore recommended that the article be retracted.

Bernadett Tildy, Alberto Papi, Paolo Casolari, Gaetano Caramori, Karen Limbert Rempel, Andrew J. Halayko, Ian Adcock and Kian Fan Chung agree with this retraction. Mark Perry has not responded to correspondence from the Publisher about this retraction.
